# Stuck in transition - admission to the neonatal intensive care unit after shoulder dystocia: analysis of risk factors and assessment of postnatal care

**DOI:** 10.1186/s40748-025-00244-5

**Published:** 2026-01-07

**Authors:** Alexandros Rahn, Carolin Böhne, Bettina Bohnhorst, Sabine Pirr, Corinna Peter, Svea Kleiner, Charlotte Mindermann, Patrick Galland, Vivien Dütemeyer, Constantin von Kaisenberg, Lars Brodowski

**Affiliations:** 1 Department of Pediatric Pulmonology, Allergology and Neonatology, Hannover Medical School, Hannover, Germany; 2 Department of Pediatric Cardiology and Pediatric Intensive Care, Hannover Medical School, Hannover, Germany; 3 Department of Pediatric Kidney, Liver and Metabolic Diseases, Hannover Medical School, Hannover, Germany; 4 Department of Child and Adolescent Psychiatry, Children’s Hospital Auf der Bult, Hannover, Germany; 5 Department of Obstetrics and Gynecology, Hannover Medical School, Hannover, Germany

**Keywords:** Shoulder dystocia, Neonatology, Obstetrics, Cardiopulmonary resuscitation, Birth injury

## Abstract

**Background:**

This study aimed to assess frequency and causes of neonatal intensive care unit (NICU) admissions in neonates with shoulder dystocia (SD).

**Methods:**

A retrospective analysis of 116 SD cases at a tertiary perinatal center was performed (2007 – 2023). Maternal and neonatal parameters were evaluated in relation to NICU admission.

**Results:**

Seventeen neonates (14.7%) were admitted to the NICU. All showed respiratory distress; five required cardiopulmonary resuscitation, two of them received therapeutic hypothermia for hypoxic-ischemic encephalopathy. Compared to non-admitted infants, NICU neonates had significantly lower umbilical artery pH and base deficit, lower Apgar scores, and higher rate of birth injuries. They also had a significantly longer median head-to-body delivery interval (HBDI, 5.0 vs. 2.0 minutes, *p* < 0.0001). No neonatal deaths occurred before discharge.

**Conclusion:**

SD can lead to severe postnatal adaptation problems in newborns, requiring timely and structured interdisciplinary management. Our findings suggest that a HBDI of ≥ five minutes serve as a clinically applicable parameter to identify neonates at increased risk of NICU admission and appears to be associated with a higher likelihood of brachial plexus injury. Furthermore, greater fetal size, reflected by higher birth weight and body surface area, was related to the occurrence of birth injuries overall, underscoring the relevance of both extraction time and fetal dimensions for postnatal outcomes after SD.

## Background

Shoulder dystocia (SD) is a rare but serious obstetric complication, occurring in approximately 0.7% of all births. It is associated with perinatal asphyxia, skeletal injuries, brachial plexus injury (BPI), and leads to infant death in approximately 0.4% − 0.5% of cases [1–3]. Maternal complications may include uterine rupture, urethral injuries, and third-or fourth-degree perineal tears [4]. While definitions vary, SD is commonly diagnosed when the infant’s head is deeply retracted into the vulva and the shoulders do not deliver with gentle traction or within one minute after the head [5, 6]. The likelihood of SD increases with birth weight, making macrosomia the strongest known risk factor; approximately 60% of affected neonates weigh more than 4 kg [7–9]. Maternal diabetes - preexisting or gestational - is a major contributing factor, since SD occurs four to five times more frequently in infants of diabetic mothers compared to weight-matched neonates of non-diabetic mothers, likely due to altered fetal body composition [10–12]. Additional risk factors include operative vaginal delivery, maternal obesity, excessive gestational weight gain, post-term pregnancy, multiparity, male sex, and maternal pelvic anomalies [13–16].

BPI occurs in 4.7% to 15% of affected neonates, most often involving the upper plexus („Erb-Duchenne“), which typically resolves in about 80% of cases within the first six months. Clavicle fractures (5% − 23%) are the most common skeletal injury, while humeral fractures (~6%) are less frequent and usually occur during maneuvers such as internal rotation or posterior arm extraction [17–19]. During labor arrest, impaired umbilical cord perfusion and subsequent fetal hypoxia may occur, potentially leading to respiratory distress and asphyxia. The likelihood of perinatal acidosis and hypoxemic-ischemic encephalopathy (HIE) increases significantly when the head-to-body delivery interval (HBDI) exceeds five minutes [18].

In general, a range of perinatal complications may necessitate neonatal intensive care unit (NICU) admission in term neonates, including respiratory distress, congenital anomalies, cardiovascular instability, hypoglycemia, low Apgar scores, perinatal acidosis, infection, or meconium aspiration [20]. Given the potential for several of these conditions to arise in the context of SD, it is essential to better understand the specific factors leading to NICU admission in affected neonates.

This study retrospectively analyzes a 17-year cohort of neonates affected by SD at a tertiary perinatal center, with a focus on the frequency and causes of NICU admission. Currently, there is limited evidence describing the characteristics and postnatal condition of infants requiring NICU admission after SD. Therefore, by evaluating relevant clinical and laboratory parameters, we sought to identify factors associated with early neonatal compromise in this population.

## Methods

### Study design and settings

This study is a retrospective cohort study conducted at a tertiary perinatal center in northern Germany, which manages approximately 2.500 – 3.000 deliveries per year. All deliveries are attended by a midwife and an obstetrician, with a senior obstetrician available in-house at all times. In higher-acuity situations, including abnormal intrapartum events or anticipated complications, the senior obstetrician is immediately called to the delivery room. In the event of SD, both the anesthesiology team and a neonatologist are additionally alerted and attend the delivery according to the institutional emergency protocol. The level III NICU comprises 23 beds, including 12 intensive care beds with invasive ventilation capability and 11 intermediate care beds. On the maternity ward, neonates are either roomed-in with their mothers or monitored in a separate five-bed pediatric observation unit (POU), established in 2009 and staffed by pediatric nurses. A pediatrician is present on site during daytime hours and available on call from the NICU at all other times. This area is intended for newborns who require closer clinical observation but do not necessitate NICU-level care, such as those with a gestational age ≥34 ^0^/_7_ weeks, transient postnatal adaptation disorders, or other conditions requiring short-term monitoring (e.g., phototherapy due to hyperbilirubinemia or glucose infusion). Infants requiring any form of respiratory support are not eligible for care in the POU and are admitted directly to the NICU.

### Participants and data sources

All births between 2007 and 2023 were screened for the ICD-10 code O66.0 (obstructed labor due to SD) and exported to Microsoft^®^ Excel (version 16.90, Washington, USA). Systematic electronic ICD-10 documentation was introduced at our institution in 2007, which defined the start of the study period. Medical records were reviewed by two clinical experts (AR and LB).

Based on the electronic medical records, maternal parameters such as age, pre-pregnancy body mass index (BMI), number of pregnancies and diabetes status (preexisting or pregnancy-related) were recorded for each birth. Neonatal parameters assessed included, among others, Apgar scores (at 1, 5, and 10 minutes), umbilical artery pH and base deficit (UA-pH, UA-BD), and documented reasons for NICU admission. For the purpose of this analysis, respiratory distress was defined as the need for supplemental oxygen beyond 10 minutes after birth, use of delivery room CPAP or invasive ventilation, clinically observed irregular breathing patterns (e.g., grunting, nasal flaring, chest retractions), or arterial hypercapnia (pCO₂ > 60 mmHg). Perinatal acidosis was defined as an UA-pH < 7.1.

Because this study represents a retrospective full-cohort analysis including all available cases of SD during this period, no a priori sample size calculation was performed, and a formal power analysis for the primary outcome (NICU admission) was not applicable. The study was approved by the Ethics Committee of Hannover Medical School (No. 11894-BO-K-2025) and conducted in accordance with the Declaration of Helsinki.

### Variables and outcomes

The primary outcome was admission to the NICU following SD. Secondary outcomes included the occurrence of birth injury (overall and by type), the HBDI, Apgar scores at 1, 5 and 10 minutes, UA-pH and UA-BD, signs of respiratory distress, and the need for delivery-room cardiopulmonary resuscitation (CPR). Maternal variables included age, pre-pregnancy body mass index (BMI), parity, and diabetes status (pre-existing or gestational). Neonatal variables included birth weight, birth length, birth head circumference, and body surface area (BSA).

### Statistical analysis

All statistical analyses were performed using GraphPad Prism 10.4.1 (GraphPad Software, Boston, MA, USA). Following the Shapiro-Wilk normality test, data were presented as mean ± standard deviation or median and range, as appropriate. For comparison between groups (NICU-admitted vs. non-admitted), either the t-test or the Mann-Whitney U-test was applied. Categorical variables were compared using Fisher’s exact test. Potential risk factors for NICU admission were assessed through groupwise comparison of clinical and laboratory parameters. In a subgroup analysis, neonates who remained on the maternity ward were further stratified by level of postnatal care (POU vs. rooming-in) and compared accordingly. A p-value < 0.05 was considered statistically significant.

## Results

**Fig. 1:**
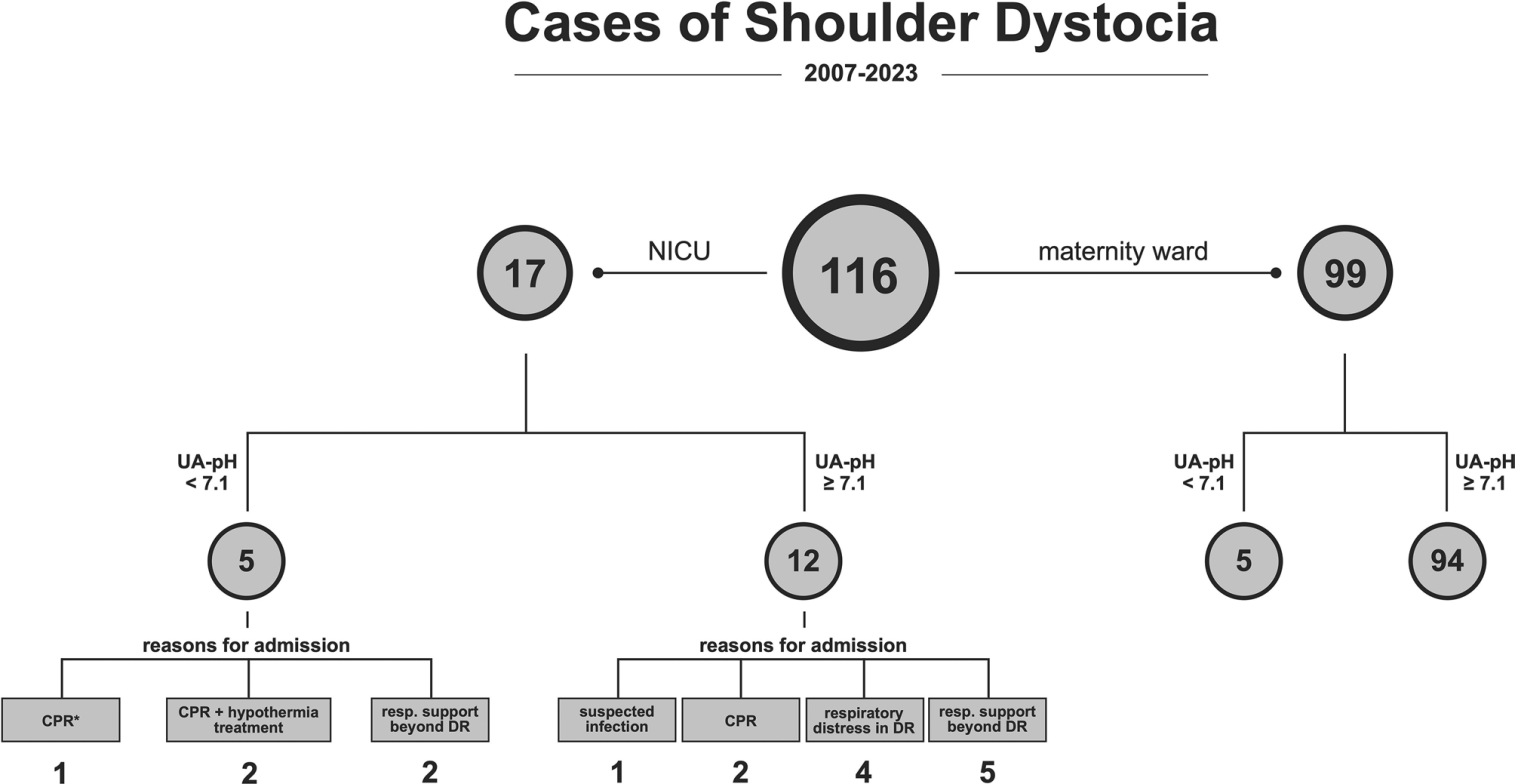
Flowchart illustrating the distribution of 116 cases of shoulder dystocia by NICU admission and umbilical artery pH (UA-pH), with documented reasons for NICU admission. CPR: cardiopulmonary resuscitation, DR: delivery room, NICU: neonatal intensive care unit. *Unlike all other CPR cases, this neonate remained free of respiratory support beyond the delivery room

Out of 41,256 live births between 2007 and 2023, 116 cases of SD were identified (incidence: 0.28%), all of them in singleton deliveries. Of these, 17 (14.7%) were admitted to the NICU, 39 (33.6%) remained in the POU at the maternity ward, and 57 (49.1%) remained with their mothers (rooming-in). For three infants, the location of care could not be determined retrospectively. Figure 1 provides a structured overview of the SD cohort, with both NICU-admitted and non-admitted neonates stratified by UA-pH. For the NICU group, documented reasons for admission are additionally shown.

As shown in Tables 1 and 2, there were no significant differences in maternal characteristics or neonatal somatic parameters between NICU-admitted and non-admitted neonates. All deliveries were either spontaneous vaginal deliveries or assisted by vacuum extraction, with no significant differences in delivery mode between the NICU and non-NICU groups.

**Table 1:** Maternal characteristics for the total cohort and stratified by NICU admission status

Maternal characteristics				
Characteristic	Total (*n* = 116)	NICU admitted (*n* = 17)	Non-admitted (*n* = 99)	*p*-value
Maternal age (years)	32.5 (5.5)	31.88 (5.34)	32.58 (5.57)	0.6342†
Pre-pregnancy BMI (kg/m^2^)	25.3 (16.6 - 47.9), *n* = 108	26.7 (21.8 - 47.9)	24.6 (16.6 - 45), *n* = 91	0.2609‡
Number of pregnancies, incl. Current	2 (1 - 14)	2 (1 - 6)	2 (1 - 14)	0.3264‡
Obstetric interventions	Mediolat. episiotomy: 43/116 (37.1%)	Mediolat. episiotomy: 10/17 (58.8%)	Mediolat. episiotomy: 33/99 (33.3%)	0.058§
	Symphyseal disruption 1/116 (0.86%)	Symphyseal disruption 1/17 (5.8%)	Symphyseal disruption 0/99 (0%)	0.147§
Blood loss at birth (ml)	250 (150 - 1300), *n* = 92	300 (200 - 600), *n* = 11	250 (150 - 1300), *n* = 81	0.2172‡
Any form of mothers diabetes	25/116 (21.6%)	5/17 (29.4%)	20/99 (20.2%)	0.522§

Total cohort: n = 116 (NICU admitted, n = 17; not admitted, n = 99); missing data are indicated where applicable. Values are presented as mean (SD) for normally distributed data and as median (range) for non-normally distributed data. Comparisons were performed between mothers of NICU admitted and non-admitted neonates. Statistical tests used: † unpaired t-test, ‡ Mann-Whitney U-test, § Fisher’s exact test. p < 0.05 was considered statistically significant

**Table 2:** Neonatal clinical and laboratory characteristics for the total cohort and stratified by NICU admission status

Neonatal characteristics				
Characteristic	Total (*n* = 116)	NICU admitted (*n* = 17)	Non-admitted (*n* = 99)	*p*-value
Birth at term	114/116 (98.3%)	17/17 (100%)	97/99 (98%)	1§
Sex of neonate	Female: 57 (49.1%)	Female: 10 (58.8%)	Female: 47 (47.5%)	0.439§
	Male: 59 (50.9%)	Male: 7 (41.2%)	Male: 52 (52.5%)	
Gestational age at birth (p.m., weeks)	40.1 (36.0 - 42.0)	39.7 (38.0 - 41.1)	40.3 (36.0 - 42.0)	0.23‡
Mode of delivery	Vaginal delivery: 96/116 (82.8%)	Vaginal delivery: 13/17 (76.5%)	Vaginal delivery: 83/99 (83.8%)	0.49§
	Vacuum extraction: 20/116 (17.2%)	Vacuum extraction: 4/17 (23.5%)	Vacuum extraction: 16/99 (16.2%)	
Birth weight (kg)	4.1 (2.7 - 5.6)	4.2 (3.2 - 4.7)	4.1 (2.7 - 5.6)	0.9245‡
Birth length (cm)	54 (46 - 60)	54 (46 - 57)	54 (49 - 60)	0.7125‡
Birth head circumference (cm)	36 (30.5 - 39)	35.3 (30.5 - 37.5)	36 (32 - 39)	0.0911‡
Birth body surface area (m^2^)	0.25 (0.19 - 0.3)	0.25 (0.2 - 0.26)	0.25 (0.19 - 0.3)	0.8670‡
Head-to-body delivery interval (min)	2.5 (1 - 9), *n* = 112	5 (2 - 9), *n* = 16	2 (1 - 8), *n* = 96	**< 0.0001**‡
Apgar 1’	7 (0 - 10)	3 (0 - 8)	8 (3 - 10)	**< 0.0001**‡
Apgar 5’	9 (2 - 10)	6 (2 - 9)	9 (4 - 10)	**< 0.0001**‡
Apgar 10’	10 (5 - 10)	8 (5 - 9)	10 (6 - 10)	**< 0.0001**‡
Umbilical artery pH	7.22 (0.09)	7.16 (0.08)	7.23 (0.09)	**0.0027**†
Umbilical artery lactat (mmol/l)	5.5 (1.8 - 11.5), *n* = 71	6.8 (4.1 - 9.9), *n* = 10	5.3 (1.8 - 11.5), *n* = 61	0.0732‡
Umbilical artery base deficit (mmol/l)	−5.7 (−16.4 - 1.2), *n* = 115	−9.1 (−16.4 - −1.7)	−5.4 (−15.2 - 1.2), *n* = 98	**0.0042**‡
Postnatal respiratory distress	55/116 (47.4%)	17/17 (100%)	38/99 (38.4%)	**< 0.0001**§
Cardiopulmonal resuscitation	5/116 (4.3%)	5/17 (29.4%)	0/99 (0%)	**< 0.0001**§
Any birth injury	20/116 (17.2%)	7/17 (41.2%)	13/99 (13.1%)	**0.01**§

Total cohort: n = 116 (NICU admitted, n = 17; not admitted, n = 99); missing data are indicated where applicable. Values are presented as mean (SD) for normally distributed data and as median (range) for non-normally distributed data. Comparisons were performed between NICU admitted and non-admitted neonates. Statistical tests used: † unpaired t-test, ‡ Mann-Whitney U-test, § Fisher’s exact test. p < 0.05 was considered statistically significant and is shown in bold

Birth injuries occurred in 20/116 newborns (17.2%), with multiple injuries documented in four cases. The most common were upper BPIs (*n* = 17), followed by clavicle fractures (*n* = 3), proximal radius fracture (*n* = 1) and one neonate with a combination of humerus fracture, dissections of the brachiocephalic trunk and left internal carotid artery, as well as bilateral adrenal hemorrhage. Overall, 41.2% (7/17) of neonates admitted to the NICU and 13.1% (13/99) of those not admitted had a birth injury (*p* = 0.01). A prolonged HBDI (≥5 min) was significantly associated with upper BPI (6/15 [40%] vs. 10/97 [10.3%]; OR 5.8; *p* = 0.008), and greater fetal size with the occurrence of any birth injury (birth weight: median 4.34 kg vs. 4.03 kg; *p* = 0.008; BSA: median 0.255 m^2^ vs. 0.250 m^2^; *p* = 0.02). Of the 13 neonates with birth injuries who remained on the maternity ward, 11 had isolated upper BPI, one had a clavicle fracture, and one presented with both.

All NICU-admitted neonates exhibited respiratory distress in the delivery room, and five were additionally diagnosed with perinatal acidosis. Among the 17 admitted neonates, five required CPR according to the European Resuscitation Council (ERC) neonatal guidelines [21] (median duration: 2.0 minutes), which included positive pressure ventilation, supplemental oxygen, and volume expansion as indicated. Four of these infants were subsequently intubated in the delivery room due to persistent respiratory insufficiency. Both neonates who later received hypothermia treatment for moderate to severe HIE were among this group. While eleven neonates required ongoing respiratory support beyond initial stabilization (five invasive, six non-invasive), others were admitted for monitoring due to impaired postnatal adaptation with respiratory distress in the delivery room or suspected infection (Fig. 1). The length of stay in the NICU varied greatly, with a median duration of 3.5 days (range: 1 - 35). No substantial difference in the duration of NICU stay was observed between neonates requiring invasive ventilation and those receiving either non-invasive respiratory support or no respiratory support. Of note, all twelve neonates without invasive ventilation during their NICU stay had been stabilized with CPAP in the delivery room.

Beyond treatment interventions, differences in immediate postnatal condition were also reflected by significantly lower Apgar scores at 1, 5, and 10 minutes in neonates admitted to the NICU compared to those who remained in the maternity ward (*p* < 0.0001 each). Detailed analyses further showed that lower UA-pH and UA-BD were significantly associated with NICU admission (Table 2). An UA-pH < 7.1 was documented in 5/17 to NICU admitted infants (29.4%) but only in 5/99 neonates (5.1%) who remained in the maternity ward. Notably, these five non-admitted neonates with perinatal acidosis showed no signs of birth injury, had a 5-minute Apgar score ≥ 7, and demonstrated normalization of blood gas analysis within the first two hours of life.

**Fig. 2:**
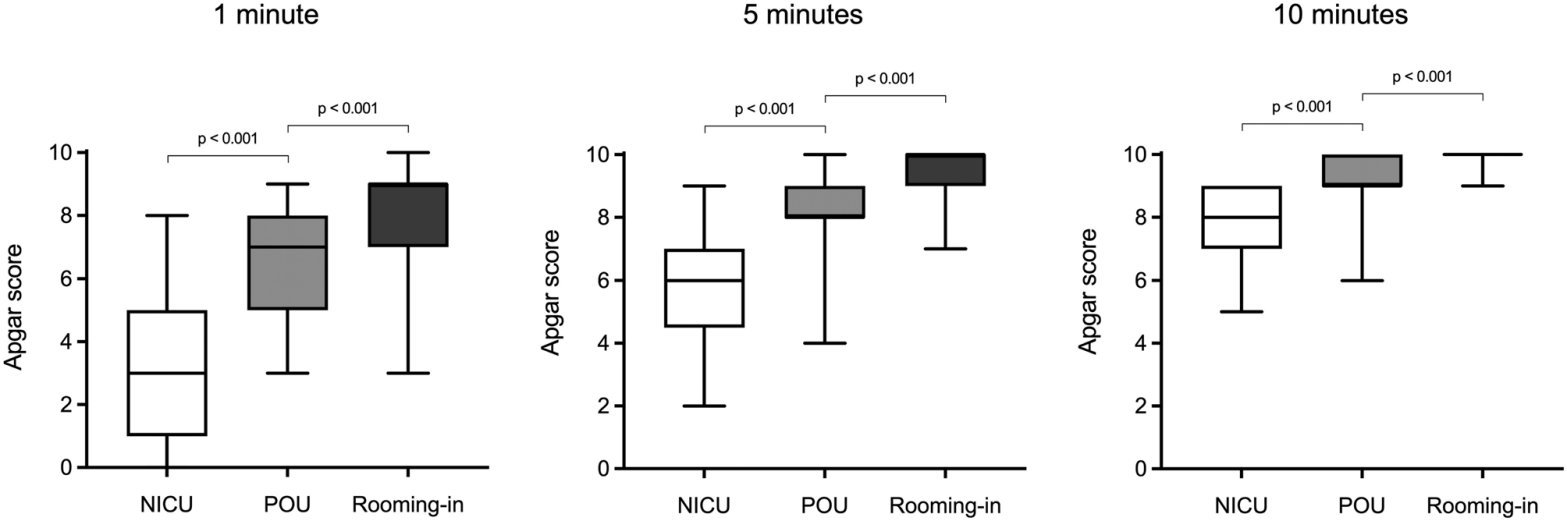
Apgar scores at 1, 5, and 10 minutes for NICU (*n* = 17), POU (*n* = 39), and rooming-in neonates (*n* = 57). Boxes represent the interquartile range (25th − 75th percentile); black lines indicate the median. Whiskers extend from minimum to maximum values. Statistical comparisons were performed using the Mann-Whitney U-test with Bonferroni correction for two pairwise comparisons (NICU vs. POU, POU vs. Rooming-in); *p* < 0.025 was considered statistically significant. NICU: neonatal intensive care unit, POU: pediatric observation unit

Of the 99 neonates who were not admitted to the NICU, 57 remained in rooming-in with their mothers, while 39 were transferred to the POU within the maternity ward for continuous monitoring. In three cases, the level of postnatal care could not be determined due to incomplete documentation. Compared to rooming-in neonates, those assigned to the POU had significantly lower Apgar scores at 1, 5, and 10 minutes (*p* < 0.001 each, Fig. 2), while UA-pH and UA-BD, as well as somatic parameters did not differ significantly. CPAP support in the delivery room was significantly more frequent in the POU group than in the rooming-in group (69.2% vs. 19.3%, *p* < 0.001), but none of the neonates who remained on the maternity ward required respiratory support beyond the initial stabilization phase. Comparison between the POU and NICU groups showed significantly lower Apgar scores (*p* < 0.001 each), lower UA-pH (*p* = 0.034), and more negative UA-BD values (*p* = 0.047) in NICU-admitted neonates.

**Fig. 3:**
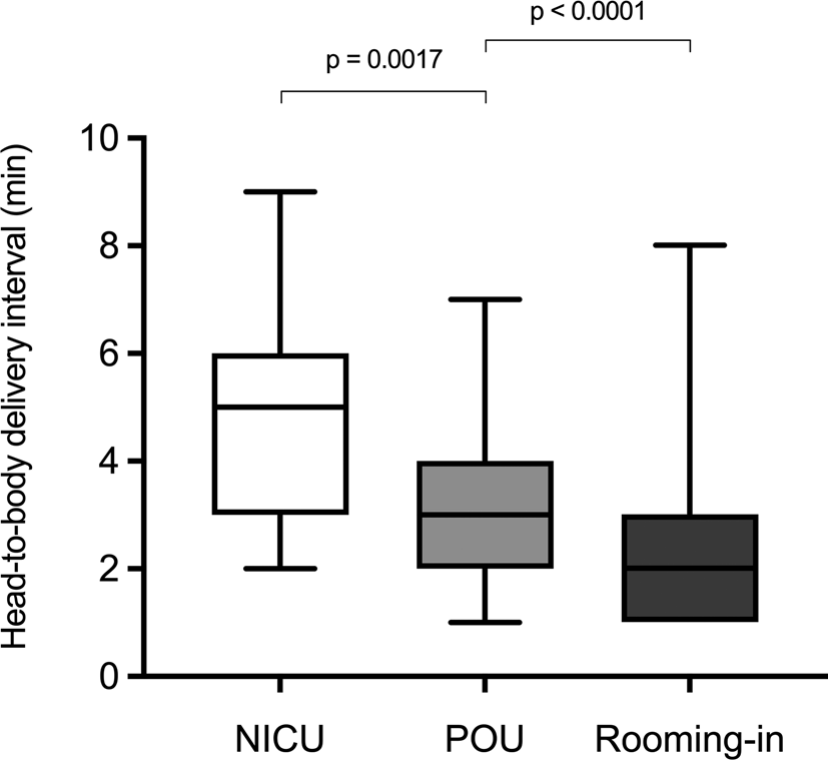
HBDI for NICU (*n* = 16), POU (*n* = 39), and rooming-in neonates (*n* = 57). Boxes represent the interquartile range (25th − 75th percentile); black lines indicate the median. Whiskers extend from minimum to maximum values. Statistical comparisons were performed using the Mann-Whitney U-test with Bonferroni correction for two pairwise comparisons (NICU vs. POU, POU vs. Rooming-in); *p* < 0.025 was considered statistically significant. NICU: neonatal intensive care unit, POU: pediatric observation unit, HBDI: head-to-body delivery interval

The median HBDI was longer in neonates admitted to the NICU compared with those admitted to the POU (5.0 vs. 3.0 min; *p* = 0.0017), whereas the median HBDI in the POU exceeded that in rooming-in neonates (3.0 vs. 2.0 min; *p* < 0.0001, Fig. 3). Neonates requiring CPAP in the delivery room had a longer median HBDI compared with those without respiratory support (3.5 vs. 2.0 min; *p* < 0.0001). ROC analysis demonstrated good discriminatory ability of the HBDI for NICU admission (AUC 0.82). A threshold of ≥ 5 minutes yielded 56% sensitivity and 94% specificity (data not shown).

## Discussion

This study provides a comprehensive analysis of NICU admissions following SD over a 17-year period at a tertiary university hospital in northern Germany. Respiratory distress, metabolic acidosis, low Apgar scores, and the need for CPR emerged as key determinants of NICU admission. By analyzing postnatal management across different levels of care, this study provides clinically relevant insights into early adaptation patterns and practical decision-making following SD.

Previous studies have shown that maternal diabetes and obesity of the mother increase the risk of SD, largely due to altered fetal growth and a higher incidence of macrosomia [7, 11, 22]. In our cohort, we observed a tendency toward a higher proportion of maternal diabetes and elevated pre-pregnancy BMI in the NICU group, although neither factor reached statistical significance. Birth weight was similarly high in both groups and also showed no significant difference. These findings suggest that maternal metabolic factors affecting fetal growth and birth weight may not fully explain the observed differences in neonatal condition, suggesting the influence of additional contributing factors not fully captured by our analysis. However, given the limited sample size, these results should be interpreted with caution.

In our study, the NICU admission rate following SD was 14.7%. To our knowledge, the only comparative report on NICU admissions due to SD is provided in a multi-center study that observed an admission rate of 9.5% based on data from 19 U.S. hospitals [23]. While both analyses were conducted in level III perinatal centers, Mendez-Figueroa’s rate reflects a multi-center average, whereas our data represent a single-center outcome, which could account for differences in clinical thresholds, patient selection, or local admission practices. In our cohort, mothers were generally older, more frequently affected by diabetes, and their neonates were both heavier and more often delivered at or beyond 40 weeks’ gestation. These are well-established perinatal risk factors that may increase the likelihood of SD-associated morbidity and compromise postnatal adaptation [12, 24]. In line with this, we observed higher rates of 5-minute Apgar scores < 7 (10.3% vs. 3.2%, *p* < 0.001) and birth injuries (17.2% vs. 9.9%, *p* = 0.017). This constellation likely contributed to the increased frequency of NICU admissions in our population. However, as a single-center retrospective study with a limited sample size, our findings should be interpreted with caution, and direct comparison to multi-center datasets must be considered exploratory rather than confirmatory.

Birth injuries were observed in 20 neonates (17.2%), with upper BPIs accounting for 17 cases (14.7%), which lies within the reported range of 4.7% − 15% for SD-associated deliveries. Clavicle fractures occurred in only 2.6% of cases, notably below previously reported rates (5% − 23%) [19]. This discrepancy may reflect differences in patient selection, diagnostic criteria, the threshold for imaging-based confirmation, or refinements in obstetric techniques - particularly in the application of traction and specific delivery maneuvers [25]. Additionally, individual variations in fetal positioning and maternal pelvic anatomy may have facilitated delivery in some cases, thus reducing the need for excessive traction and, consequently, lowering the incidence of clavicle fractures.

In line with previous reports [26, 27], a prolonged HBDI (≥5 min) was significantly associated with upper BPI, and greater fetal size (reflected by higher birth weight and BSA) with the occurrence of any birth injury. These results confirm the persistent relevance of extraction time and fetal size as determinants of neonatal outcome in SD. The significant association between birth injuries and NICU admission likely reflects the overall burden of perinatal compromise rather than injury as the primary admission reason. While none of the neonates required specific treatment for birth injury alone, our findings suggest that in cases of postnatal adaptation disorders, targeted screening for delivery-related injuries may be warranted to avoid underdiagnosis during the initial recovery phase.

As expected, respiratory distress was the most common reason for NICU transfer, consistent with previous reports on the impact of perinatal hypoxia in SD [28]. Perinatal hypoxia can impair the normal transition from fetal to neonatal respiration through mechanisms such as delayed lung fluid clearance and reduced pulmonary blood flow [29]. This can result in transient respiratory distress, typically presenting as irregular breathing, tachypnea, hypoxemia, or hypercapnia shortly after birth [30]. All neonates admitted to the NICU had shown signs of respiratory distress during immediate postnatal care. Those who did not require invasive ventilation during their stay had been stabilized with CPAP in the delivery room. These findings suggest that respiratory distress following SD was predominantly transient and consistent with post-hypoxic adaptation rather than primary pulmonary pathology. Early delivery room interventions, particularly timely application of CPAP, may have contributed to stabilization and potentially limited the need for prolonged respiratory support in some cases. However, the use of CPAP in term neonates should be weighed carefully, as early CPAP could be associated with an increased risk of pneumothorax in this population [31–33].

In addition to respiratory adaptation issues, perinatal acidosis was frequently observed across the cohort. While the reported incidence of UA-pH < 7.1 in the overall obstetric population ranges from 1.1% − 3.4% [34, 35], our SD-cohort exhibited a notably higher rate of 8.6% (10/116), supporting previous findings that SD increases the risk of perinatal acidosis [36]. Importantly, our study confirms that lower UA-pH and UA-BD values are significantly associated with NICU admission, reinforcing their role as crucial early indicators of neonatal distress [37]. The severity of intrapartum compromise was further evident in five neonates who required immediate CPR at birth. Two of them received hypothermia therapy for clinically diagnosed moderate to severe HIE, reflecting the potential for hypoxic-ischemic brain injury following prolonged or traumatic deliveries [38]. Therapeutic hypothermia is a key component of postnatal management in neonates with moderate to severe HIE and has been shown to improve neurological outcomes [39–41]. In both infants, follow-up cranial magnetic resonance imaging revealed no hypoxic brain injury, and neurological status at discharge was unremarkable. Both were subsequently referred to a social pediatric center for clinical follow-up examinations.

Beyond these established laboratory and clinical indicators, the duration of the HBDI emerged as an additional factor closely associated with neonatal condition in our cohort. In our study, the HBDI was significantly longer in neonates admitted to the NICU than in those managed in the POU or rooming-in. ROC analysis demonstrated good discriminatory ability (AUC 0.82), with a threshold of ≥ 5 minutes providing 56% sensitivity and 94% specificity. These findings suggest that the HBDI, a parameter readily available at birth, may serve as a clinically applicable parameter to identify neonates at increased risk of postnatal adaptation disorders and need for intensive care. Previous studies have examined associations between HBDI and biochemical as well as neurological outcomes, with partly divergent findings: One analysis found no linear relationship with UA-pH [42], whereas another demonstrated a minute-by-minute decline in UA-pH and a sharply increased risk of severe acidosis (UA-pH < 7) and HIE beyond five minutes [43]. Another study further showed that neonates with BPI and neonatal depression had substantially longer HBDI than uncomplicated cases, with more than half delivered after four minutes [26]. Our study is to our knowledge the first to demonstrate a direct association between prolonged HBDI and the need for NICU admission. This extends the existing evidence from physiological and surrogate measures to a clinically meaningful endpoint and highlights that an HBDI approaching or exceeding five minutes should raise concern for compromised adaptation and may justify early escalation of care.

In light of these findings, structured and interdisciplinary emergency protocols should be an integral part of care for neonates at risk of severe perinatal complications requiring CPR or other intensive postnatal interventions. Simulation-based team training and standardized algorithms that enable early identification of fetal distress and coordinated emergency preparation have been shown to improve both neonatal resuscitation effectiveness and neurological outcomes [44–46].

A distinct subgroup of neonates in our cohort required clinical monitoring due to impaired postnatal adaptation but did not necessitate intensive care. These infants received continuous observation in an POU within the maternity ward, staffed by pediatric nurses with neonatal experience. A physician from the NICU was available on-site at all times. The majority of POU-assigned neonates showed respiratory distress in the delivery room and had lower Apgar scores than those in rooming-in care, indicating a need for temporary enhanced surveillance. However, in comparison to NICU-admitted neonates, POU infants had notably better clinical parameters, suggesting a lower degree of postnatal compromise and justifying care outside the intensive care setting.

While the POU model has been described as a potential means to optimize neonatal triage and resource use [47], our retrospective data do not allow conclusions on its specific impact. Future prospective studies with standardized criteria are needed to evaluate the role of POUs in the management of neonates after SD and to explore their broader function within modern perinatal systems, including potential clinical, emotional, and logistical benefits.

### Strengths and limitations

A particular strength of this study is the detailed documentation of both maternal and neonatal clinical data, enabling a differentiated analysis of factors associated with NICU admission. The long study duration of 17 years allowed for the accumulation of a relevant case number despite the rarity of SD. Furthermore, the setting in a tertiary perinatal center with structured care pathways and interdisciplinary collaboration ensures a high degree of diagnostic accuracy and consistency in perinatal management, supporting the relevance of findings to similar institutions. Importantly, the study design included not only NICU-admitted neonates but also those who remained on the maternity ward, enabling a more comprehensive assessment of postnatal care stratification and clinical decision-making across different levels of care.

Some aspects of the study design should be considered when interpreting the results. As a single-center analysis, generalizability to other care environments may be limited. The retrospective design is subject to potential documentation bias and limits control over confounding variables. In addition, the long observation period may have introduced variability due to temporal changes in obstetric and neonatal management practices, including advances in delivery room resuscitation and evolving criteria for NICU admission. Finally, the absence of long-term follow-up data precludes conclusions regarding potential developmental or neurological outcomes beyond discharge. Future prospective studies with standardized follow-up are needed to address these gaps.

## Conclusion

SD can lead to severe neonatal compromise. In this cohort, NICU admission was significantly associated with postnatal respiratory distress, perinatal acidosis, low Apgar scores, the need for delivery-room CPR, and a prolonged HBDI (≥5 minutes). Additionally, greater fetal size was linked to the occurrence of birth injuries, underscoring the relevance of extraction time and fetal dimensions for early postnatal risk assessment after SD.

## Data Availability

The datasets generated and analyzed during this study are not publicly available due to patient confidentiality regulations but are available from the corresponding author upon reasonable request.

## Abbreviations

CPAP: Continuous Positive Airway Pressure
CPR: Cardiopulmonary Resuscitation
DR: Delivery Room
ERC: European Resuscitation Council
HBDI: Head-to-Body Delivery Interval
HIE: Hypoxic-Ischemic Encephalopathy
NICU: Neonatal Intensive Care Unit
POU: Pediatric Observation Unit
SD: Shoulder Dystocia
UA-BD: Umbilical Artery Base Deficit
UA-pH: Umbilical Artery pH
